# Data-Driven Bioprocess Optimization: Lipase Production by Aspergillus Niger ATCC 1004 in COCOA and Palm Oil Byproducts Based Solid State Fermentation

**DOI:** 10.1007/s12010-026-05681-2

**Published:** 2026-04-01

**Authors:** Eliézer Luz do Espirito Santo, Bruno Henrique Leão Pimentel, Igor Carvalho Fontes Sampaio, Thiago Pereira das Chagas, Erik Galvão Paranhos da Silva, Marcelo Franco, Julieta Rangel de Oliveira

**Affiliations:** 1 Biotransformation and Biocatalysis Organic Research Group, Department of Exact Sciences, Santa Cruz State University, Ilhéus, 45654-370 Brazil; 2 Department of Engineering and Computing, Santa Cruz State University, Ilhéus, 45654-370 Brazil; 3 Department of Exact Sciences, Santa Cruz State University, Ilhéus, 45654- 370 Brazil

**Keywords:** Artificial Intelligence, Bioprocess optimization, Agro-residues valorization, Lipase inducers

## Abstract

This study aimed to optimize the production of lipase via solid-state fermentation (SSF) by *Aspergillus niger* ATCC 1004, using cocoa bean shell (CBS) and palm oil sludge (POILS). An artificial neural network (ANN) integrated with a particle swarm optimization (PSO) algorithm was employed. Fermentation time (days), initial moisture (%), and POILS (%) were evaluated using a Box-Behnken design. ANN performance was assessed based on the determination coefficient (R²) and mean squared error. The PSO tested two spaces: a restricted search region (RSR, -2 to + 2) and a broader search region (BSR, -3 to + 3). The 28-neuron ANN achieved R^2^ = 0.9998 (training), R^2^ = 0.97 (testing), R^2^ = 0.91 (validation), and R^2^ = 0.97 (overall). The predicted optimal lipase yields were 621.80 U g^− 1^ (broader region, BR) and 424.43 U g^− 1^ (restricted region, RR). Experimental validation yielded 322.50 ± 4.24 U g^− 1^ (BR) and 430.00 ± 3.31 U g^− 1^ (RR). The RR demonstrated better predictive accuracy, with experimental values reaching 98.7% accuracy, optimizing the response by 150% relative to the value predicted by the quadratic model. The optimal validated conditions for SSF were 4.4 days, 48% initial moisture, and 10% POILS. These results confirm the efficiency of PSO in optimizing ANN with limited datasets, reducing additional experiments and costs. POILS proved effective in stimulating lipase secretion, offering an alternative application in the bioprocess and paving the way for future research related to production expansion, and life cycle assessment of lipase production under the identified conditions.

## Introduction

The industrial use of lipases, as an alternative to conventional chemical methods, offers significant advantages, such as processes conducted under mild temperature and pressure conditions (resulting in lower energy consumption), high specificity, and stereoselectivity. These characteristics enable the production of purer products with fewer by-products, aligning with the principles of green chemistry and sustainability [1–3]. However, the high cost of obtaining lipases remains a major obstacle to their widespread industrial application [1, 4], limiting their use to processes that generate high-value products that can offset the additional costs [5].

Lipase production on a large scale is commonly achieved through submerged fermentation (SF), which offers advantages such as greater medium homogeneity and better control over parameters like temperature and pH [6]. However, the use of refined substrates and specific nutrients, accounting for 50% of the total cost, significantly increases process expenses [5].

In recent years, solid-state fermentation (SSF) has emerged as a promising alternative for lipase production, utilizing lignocellulosic byproducts as a low-cost carbon source for microbial growth [7–9]. The primary advantage of SSF lies in its ability to use a wide variety of lignocellulosic byproducts as substrates and its lower water requirements, making the process much cheaper. Additionally, SSF promotes circular economy principles by recycling residual biomass and generating added value [5].

Different microorganisms have been employed in SSF for lipase production, including bacteria [10], filamentous fungi [11], and yeasts [12]. Among these, filamentous fungi stand out due to their superior adaptation to the low water content characteristic of SSF, enabling them to maintain metabolic activity in low-moisture environments, thus making them more efficient for enzyme production via SSF [7]. The production of fungal lipases through SSF is influenced by several factors, including temperature, fermentation time, substrate type, initial moisture, and pH [7, 13].

Studies have investigated the impact of varying these factors on enzymatic activity [14, 15], often employing either univariate approaches or multivariate designs, most notably Response Surface Methodology (RSM), which encompasses experimental strategies such as fractional factorial, full factorial, central composite, and Box-Behnken designs [16]. The univariate method, or one-factor-at-a-time (OFAT) approach, has limitations, such as requiring many experiments, increasing reagent costs, and failing to account for factor interactions. Conversely, RSM enables the simultaneous evaluation of multiple variables and their interactions through the construction of empirical models, typically second-order polynomials, fitted to experimental data [14]. These models are validated statistically, often via analysis of variance (ANOVA), and allow for the identification of optimal conditions within the defined experimental space [17]. Despite its efficiency, RSM has inherent limitations, including reliance on interpolation and restriction to the experimental domain studied, limiting their ability to predict responses beyond the minimum and maximum variable ranges [9, 15, 17].

Recently, artificial neural networks (ANN’s) have gained attention for optimizing enzyme production processes [9, 13, 18, 19]. ANN’s consist of artificial neurons organized in layers, simulating the brain’s biological learning model by adjusting connection weights through training to maximize output responses [20, 21, 18, 9, 22].

With a trained and validated model, various optimization algorithms can be applied to explore the solution space and enhance network efficiency. Among these, the particle swarm optimization (PSO) algorithm stands out for its effectiveness in identifying optimal input variable conditions that maximize the output variable while maintaining lower computational costs [20, 23]. PSO generates hundreds or thousands of input variable combinations and computes their corresponding output values based on the ANN model [24]. This approach significantly reduces the need for extensive experimental trials, which would be impractical on a large scale due to reagent consumption and high costs [21, 23]. Therefore, integrating ANN with PSO allows for efficient exploration of the solution space, optimizing results in a more practical and potentially economical approach.

Literature reports the integration of ANN’s with particle swarm optimization (PSO) algorithms (ANN-PSO) for modeling various processes, including glucose and xylose estimation in microwave-assisted pre-treatment for enzymatic hydrolysis [25], fermentation parameter optimization for coffee [26], biogas production prediction in anaerobic digesters [27], and laccase extraction from mushroom residues [28]. Despite its advantages and potential to advance bioprocess elucidation, ANN-PSO remains underexplored in the literature, particularly for optimizing SSF conditions for enzyme production.

This study hypothesizes that the amendment of lipids and fatty acids as inducers can enhance the secretion of a crude extract with lipase activity (CELA) produced by *Aspergillus niger* ATCC 1004 cultivated on lignocellulosic material. To test this hypothesis, the study aims to optimize CELA production via SSF using cocoa bean shell (CBS) as the raw material and palm oil sludge (POILS) (*Elaeis guineensis*) as a lipidic inductor employing the ANN-PSO methodology. POILS is the residue left over after the refining of palm oil [29]. In 2023, the global palm oil market was valued at USD 70.44 billion and is expected to grow at a CAGR of 5.1% between 2024 and 2030 (GVR, 2023). POILS is described as a viscous, brownish liquid, typically containing oil and grease concentrations ranging from 130 to 18,000 mg/L, along with a high content of free fatty acids (FFA) of approximately 40% [30–32].

Beyond process optimization, this study highlights the valorization of agro-industrial residues by integrating CBS and POILS within an SSF framework. The application of ANN-PSO provides a data-driven alternative to conventional optimization strategies, enabling a more efficient exploration of process variables. This combined approach contributes to the development of sustainable and economically viable bioprocesses for industrial enzyme production.

## Materials and Methods

### Data Generation for the Artificial Neural Network

#### Preparation of Inoculum

*Aspergillus niger* ATCC 1004 was obtained from the microbial collection of Fio Cruz (Rio de Janeiro, Brazil). The inoculum was prepared on potato dextrose agar (PDA) (VETEC, São Paulo, Brazil) and agar-agar (VETEC) at 28 °C for 7 days in a bacteriological incubator (Tecnal, São Paulo, Brazil). After growth, fungal spores were suspended in a Tween 80 solution (0.01%), and the spore concentration was determined using a Neubauer chamber and a microscope (Bioval, São Paulo, Brazil).

#### Solid-State Fermentation

CBS samples were provided by a cocoa processing industry located in southern Bahia, Brazil. These samples were dried in an oven (Tecnal, São Paulo, Brazil) at 50 °C for 24 h and subsequently milled in a knife mill (ACB Labor, São Paulo, Brazil) to a particle size of 2 mm. POILS (*Elaeis guineensis*) was supplied by the company Mil Select (Nilo Peçanha, Brazil) and sterilized in an autoclave at 120 °C, 1.0 atm for 15 min.

Fermentation was carried out in 125 mL Erlenmeyer flasks containing 5 g of CBS byproduct. The amount of POILS (mL/g) was calculated as a percentage based on 100% of the raw material mass (5 g of CBS, as shown in Table 1). The fermentation medium was sterilized in an autoclave (120 °C, 1.0 atm for 15 min), inoculated with 10^7^ spores g^− 1^, and the moisture content was adjusted by adding sterile distilled water. The fermentations were maintained in a bacteriological incubator (Tecnal, São Paulo, Brazil) at 30 °C. Experiments were conducted at varying moisture levels (%), time (days), and inductor concentrations (% POILS) using a Box-Behnken design with 7 levels, as detailed in Table 1.

**Table 1:** Actual and coded levels analyzed in the Box-Behnken design for lipase activity optimization in crude extract with lipase activity (CELA)

Level	Time(days)	Inductor (%)	Moisture (%)
-3	1	5	40
-2	2	10	48
-1	3	15	56
0	4	20	64
+1	5	25	72
+2	6	30	80
+3	7	35	88

**Fig. 1:**
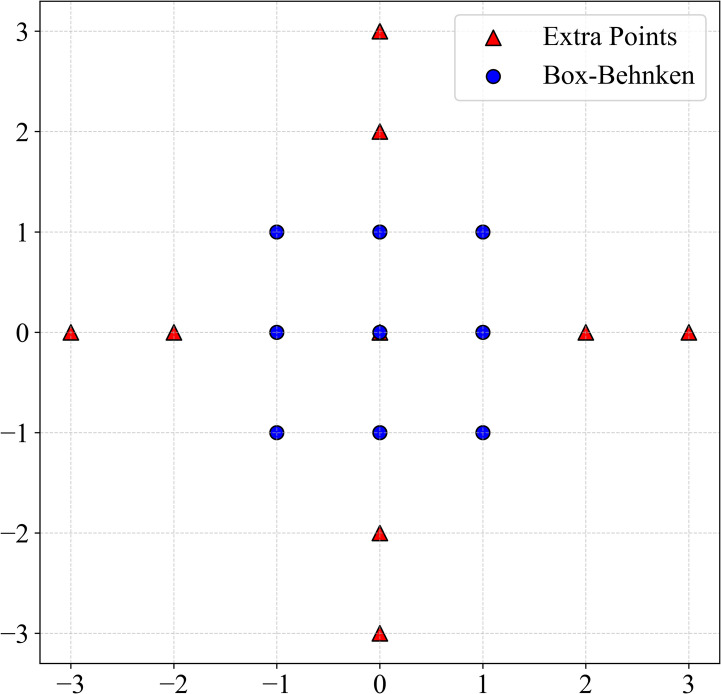
Graphical representation of the Box-Behnken design showing the experimental points for optimization of lipase activity in crude extract with lipase activity (CELA), including those used to extrapolate beyond the standard + 1 and − 1 levels

The graphical representation of the design, including the points used to extrapolate beyond the + 1 and − 1 levels of the Box-Behnken design, is shown in the Fig. 1.

#### Characterization of Substrates

The bromatological composition of the CBS samples was determined based on their contents of cellulose, hemicellulose, lignin, crude protein, lipids and ash, as well as for their neutral detergent fiber (NDF) and acid detergent fiber (ADF) fractions [33]. The results are expressed as a percentage (g/100 g) of dry matter and are presented in Table 2.

**Table 2:** Composition (% by dry weight) of the CBS sample obtained by bromatological analysis

Parameter	Composition CBS
Cellulose	20.81 ± 0.19
Hemicellulose	10.78 ± 0.84
Lignin	17.04 ± 0.51
ADF	36.62 ± 0.10
NDF	49.78 ± 0.49
Total protein	21.16 ± 0.24
Lipids	10.14 ± 0.32
Ash	1.52 ± 0.03

#### Lipase Activity Determination

After fermentation, 50 mL of distilled water was added to the 125 mL Erlenmeyer flasks. The mixture was stirred in an orbital shaker (Tecnal, São Paulo, Brazil) at 30 °C and 200 rpm for 30 min. The solution was then filtered and centrifuged (Biovera, Rio de Janeiro, Brazil). The supernatant, referred to as CELA, was used for lipase activity determination.

Lipase activity was determined using an olive oil emulsion prepared with an Arabic gum solution (3% w/w) in water and olive oil (25% w/w) as described in the literature [34]. A 5.0 mL aliquot of the emulsion, 1.0 mL of ME, and 5.0 mL of sodium phosphate buffer (0.1 M, pH 7.0) were added to a 125 mL Erlenmeyer flask and stirred in an orbital shaker (Tecnal, São Paulo, Brazil) at 37 °C and 200 rpm for 30 min. The control (blank) was prepared under the same conditions, replacing the CELA with distilled water. The reaction was stopped by adding 10 mL of ethanol (95%), and the mixture was titrated with sodium hydroxide solution (0.03 M) using phenolphthalein as an indicator. Lipase activity (LA) was calculated using the following equation:

1 $$\:LA=\frac{\left({V}_{a}-{V}_{b}\right)\times\:M\times\:1000}{t\:\times\:\:v}\:x\:\frac{ve}{gds}$$

Where *LA* is the lipase activity (U g^− 1^ of dry substrate), Va is the volume of NaOH added to the sample (mL), Vb is the volume of NaOH added to the blank (mL), *M* is the molarity of the NaOH solution, *t* is the reaction time (min), *v* is the sample aliquot volume (mL), *ve* is the total volume of the recovered extract, and gds is mass (gram) of dry substrate.

### Artificial Neural Network

#### Training the Artificial Neural Network

A feed forward Multi-Layer Perceptron (MLP) was adopted owing to its universal-approximation capability for nonlinear systems [35]. The network comprised an input layer with three neurons (fermentation time, initial moisture and POILS concentration), a single hidden layer and one output neuron representing lipase activity (LA).

Prior to training, all input variables were linearly rescaled to the interval [–1, 1] to prevent dominance of variables with larger numeric ranges and to accelerate convergence. The full data set (28 experiments) was randomly stratified into training (≈ 70%), validation (≈ 15%) and test (≈ 15%) subsets; stratification was repeated at every random initialization to guarantee representative coverage of the experimental domain.

Network parameters were learned with the Levenberg–Marquardt back-propagation algorithm (*trainlm*, MATLAB R2022a). To identify a parsimonious architecture, hidden-layer size was swept from 4 to 40 neurons. For every architecture four L-2 regularization factors (0.00, 0.10, 0.20 and 0.30) were explored, and each (architecture × regularization) setting was retrained 10 000 times from distinct random weight–bias seeds. Early stopping on the validation mean-squared error (MSE, Eq. 2) curtailed over-training.

2 $$MSE=\frac12\sum_{i=1}^n\left(y_i^{exp}-y_i^{pred}\right)^2$$

Where *n* is the number of samples, $\:{y}_{i}^{pred}$ is the predicted value, $\:{y}_{i}^{exp}$ is the experimental value.

The optimal configuration (28 hidden neurons with a regularization factor of 0.10) was selected as the candidate model for subsequent performance screening. Training a large number of networks is necessary because the limited size of the dataset results in a highly non-convex optimization landscape with numerous local minima, rendering the training process highly sensitive to initial parameter values. Consequently, a rigorous evaluation of the resulting models is required to select one with strong generalization capability.

In this study, the inputs for the ANN were fermentation time (days), lipid inductor source (%), and moisture content (%), while the output was lipase activity of crude extract (U g^− 1^) produced by *Aspergillus niger* ATCC 1004.

#### Evaluation and Selection of the Trained ANN

Because the experimental matrix is necessarily small for biological SSF studies, stringent acceptance criteria were applied to guard against optimistic bias. Candidate networks were ranked by the coefficient of determination (Eq. 3) calculated separately for the training, validation and test subsets.

3 $$\:R^2=1-\frac{{{\displaystyle\sum_{i=1}^n}\:}(y_i^{exp}{-y_i^{pred})}^2}{{{\displaystyle\sum_{i=1}^n}\:}(y_i^{exp}{-y_i^{avg})}^2}$$

Where *n* is the number of samples, $\:{y}_{i}^{pred}$ is the predicted value, $\:{y}_{i}^{exp}$ is the experimental value, and $\:{y}_{i}^{avg}$ is the mean of the experimental values.

Only models exhibiting $\:{R}_{overall}^{2}$ $\:>$ 0.96 and showing $\:\le\:$ 0.05 absolute difference between validation and test $\:{R}^{2}$ were retained. High $\:{R}_{overall}^{2}$ values may simply reflect overfitting, particularly when most of the data belong to the training set used for parameter optimization. Therefore, the network exhibiting the highest *R²* values on the validation and test sets was selected, as it is expected to offer superior generalization capability compared to a model chosen solely based on the $\:{R}_{overall}^{2}$. The best-performing ANN achieved $\:{R}^{2}$ = 0.9998 (training), 0.91 (validation), 0.97 (test) and 0.97 (overall), with an MSE of 1.43 U g^−1^ – values that indicate robust generalization despite the restricted data set.

#### Particle Swarm Optimization Algorithm

PSO is a swarm intelligence metaheuristic proposed by Kennedy and Eberhart [36]. It mimics the collective behavior of flocks of birds or schools of fish, where each individual (particle) searches for food using its own knowledge (cognitive coefficient) and the group’s knowledge (social coefficient).

In PSO, each particle represents a potential solution to the optimization problem, moving iteratively through the solution space to improve its fitness. Particles are evaluated using an objective (cost) function, and their velocities are updated based on their own best experience and the swarm’s best experience in minimizing or maximizing the cost function. The best-evaluated particle at the termination criterion is taken as the optimal solution.

In this study, PSO was executed with 50 particles for a maximum of 500 iterations or until 50 consecutive iterations showed no improvement (Table 3). Two hyper-rectangular search domains were defined:

- Restricted Search Region (RSR): 2–6 days, 10–30% POILS, 48–80% moisture (interpolative regime).
- Broader Search Region (BSR): 1–7 days, 5–35% POILS, 40–88% moisture (extrapolative regime).

The ANN served as the surrogate objective function, thus reducing wet-lab experimentation to the single set of conditions identified as globally optimal within each region.

**Table 3:** All parameters used in the particle swarm optimization (PSO) algorithm for lipase activity optimization in crude extract with lipase activity (CELA)

Configuration	Value
Software	MATLAB R2022a
Function	Particleswarm
Optimized variables	Time (days), Inductor (%), Moisture (%)
Objective function	Maximize hydrolytic activity (U g⁻¹)
Maximum iterations	500
Particle number	50
Social coefficient	2
Cognitive coefficient	2
Inertia weight range	[0.4–0.9]
Stopping criterion	Stagnation for 50 iterations
Other settings	Standard software

#### Maximization of Lipase Activity

The maximization of lipase activity was achieved through computational modeling and experimental optimization. First, the previously described methodology was used to build an ANN model that accurately represented the experiment, substituting the real experiment during optimization. PSO was then applied to the model, with each particle in each iteration representing a possible experimental setup defined by fermentation time, lipid inductor source, and moisture content. The ANN estimated the lipase activity for each setup.

Two search spaces were defined for the PSO particles: (I) A restricted search region (RSR) within [2; 6 days] for fermentation time, [10; 30%] for lipid inductor, and [48; 80%] for moisture – regions where the ANN model is expected to have higher accuracy due to interpolation; (II) A broader search region (BSR) within [1; 7 days] for fermentation time, [5; 35%] for lipid inductor, and [40; 88%] for moisture. In both regions, the PSO algorithm was executed ten times to ensure convergence to the global maximum within each region. The same procedure was repeated using a genetic algorithm (GA) to evaluate the convergence of the optimal points identified.

## Results and Discussion

### Optimization Response Surface Methodology (RSM)

The experimental points 1 to 15, corresponding to the Box-Behnken design levels (− 1, 0, + 1), were used to evaluate the predictive ability of the quadratic model (Eq. 4).

4 $$\:U\:{mL}^{-1}=\:-290.20+72.35\:T-{4.86\:T}^2-3.91\:I-{0.04\:I}^2+6.50\:M-{0.05\:M}^2+0.23\:T\:I-0.57\:T\:M+0.07\:I\:M$$

Where T is the variable Time, I is the variable Inductor and M is the variable Moisture.

**Fig. 2:**
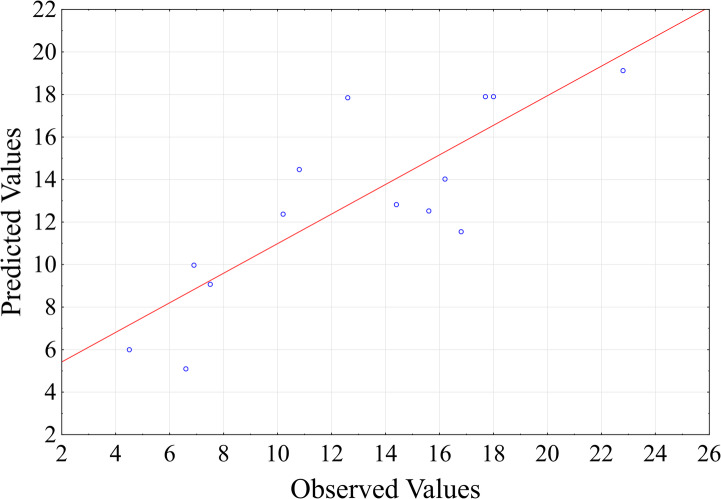
Observed vs. predicted plots for the quadratic model of lipase activity in crude extract with lipase activity (CELA) obtained from the Box-Behnken Design

**Fig. 3:**
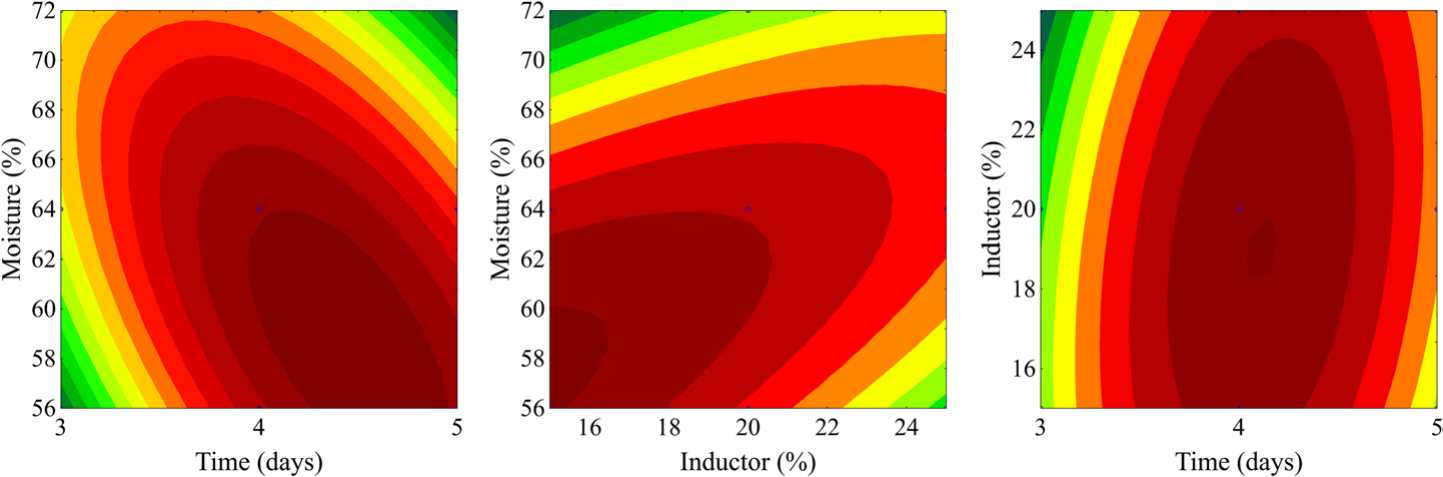
Contour graph for the optimization of lipase activity in crude extract with lipase activity (CELA) by Box-Behnken Design under SSF

The Analysis of Variance (ANOVA, Table 4) showed that the regression was not statistically significant, explaining only 70% of the data variability (R² = 0.70). The observed vs. predicted values plot is shown in Fig. 2. This limited performance highlights the need for a more complex model to better capture the system’s specificities. Nevertheless, contour plots were generated (Fig. 3), revealing an optimal region for Time around 4 days. For Moisture and Inductor, the contours suggest that the optimal region lies beyond the experimental domain, indicating that lower values may increase HA. These findings underscore the relevance of using ANN for more accurate modeling and extrapolation beyond the tested conditions.

**Table 4:** ANOVA for quadractic model for lipase activity optimization

Factor	SS^c^	df^d^	MS^e^	F	Ftab
Regression	19641.3002	9	2182.3667	1.31	4.77
Residual	8333.6991	5	1666.7398		
LOF^a^	8331.2500	3	2777.0833	2267.86	19.16
PE^b^	2.4491	2	1.2245		
Total	27974.9993	14	1998.2142		
R^2^	0.6945				

^a^Lack of Fit

^b^Pure error

^c^Sum of Squares

^d^Degrees of Freedom

^e^Mean Square

Based on the contour plot (Fig. 3), the selected conditions (4 days, 15% inductor, and 56% moisture) yielded a predicted response of 160.83 U g⁻¹. Despite the limitations of the quadratic model, the contour plots provided a preliminary estimate of the optimal region, offering insight into the system’s behavior within the experimental domain. However, given the complexity of the interactions and the indication of optimal regions beyond the tested range, a more flexible modeling approach was required [15]. Similar results were reported in a study comparing RSM and ANN, with R² values of 0.75 and 0.80, respectively [37], demonstrating the superior predictive capability of ANN. Therefore, the following section presents the results obtained using ANN modeling.

### Neural Network Architecture

The commonly used method for artificial neural networks employs MLP trained with the backpropagation algorithm. The number of neurons in the hidden layer is gradually adjusted through trial and error, evaluating model performance based on metrics such as the lowest mean squared error (MSE) and the highest coefficient of determination (R²).

**Fig. 4:**
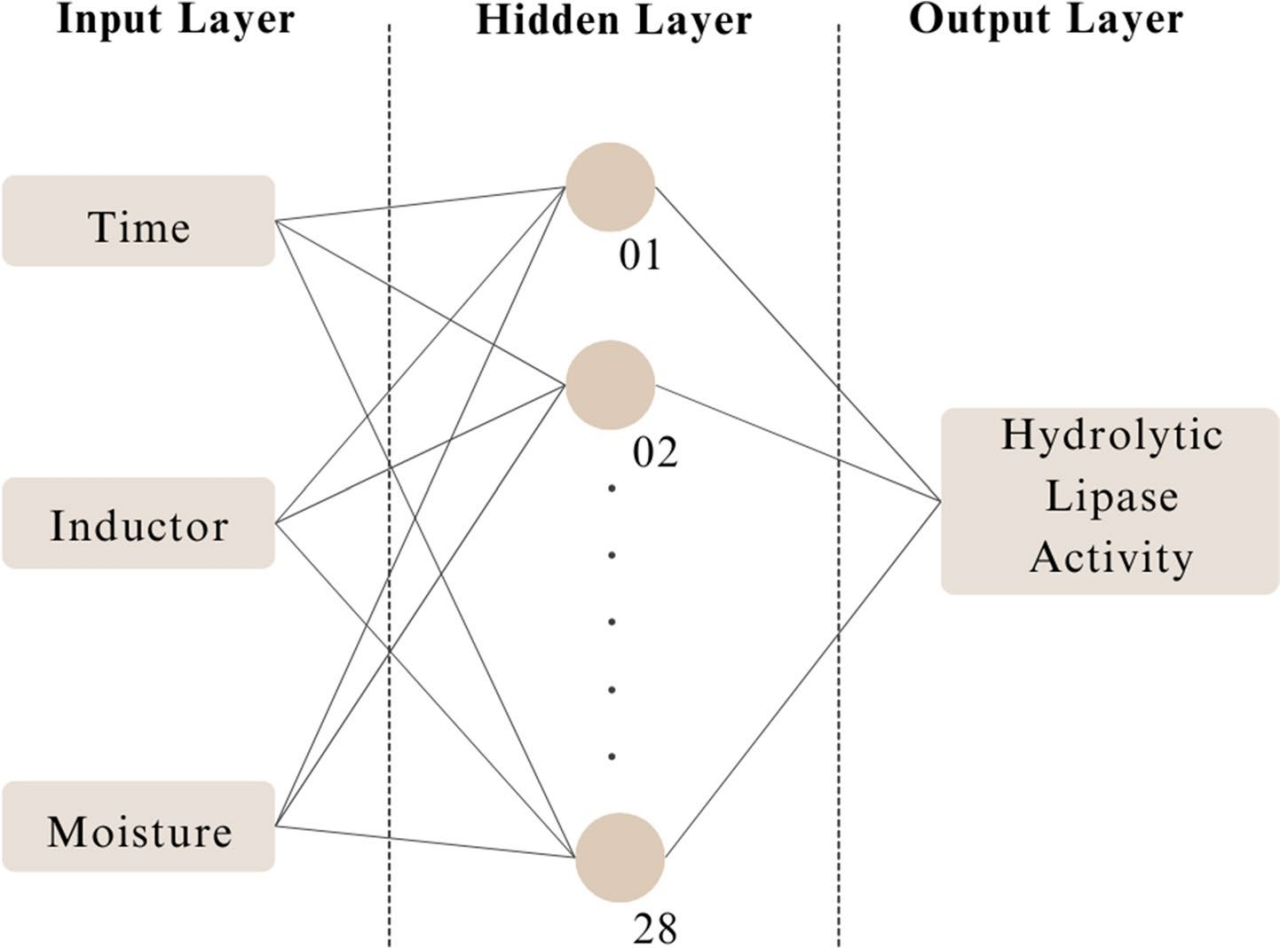
Neural network structure used to model lipase production from crude extract with lipase activity (CELA) by *Aspergillus niger* ATCC 1004 via SSF

An insufficient number of neurons may lead to underfitting (a model too simple to capture the complexities of the data), while an excessive number of neurons can cause overfitting (an overly complex model that learns noise and outliers, performing well during training but poorly on test data), compromising generalization ability. This approach aims to find a configuration that balances learning and generalization, considering the complexity of the problem and the number of available samples [9, 35]. The performance of ANN’s directly depends on the quantity and quality of the data available for training. With only 28 samples, the neural network’s ability to generalize is impaired, as limited data can lead to underfitting issues [9]. In this study, to mitigate these effects, a large number of neural networks were trained following the procedure described in Section Training the Artificial Neural Network. From this set, a neural network with 28 neurons in the hidden layer (Fig. 4) was selected based on R² values computed for the entire dataset, as well as for the validation and test sets, in accordance with the criteria outlined in Section Evaluation and Selection of the Trained ANN.

**Fig. 5:**
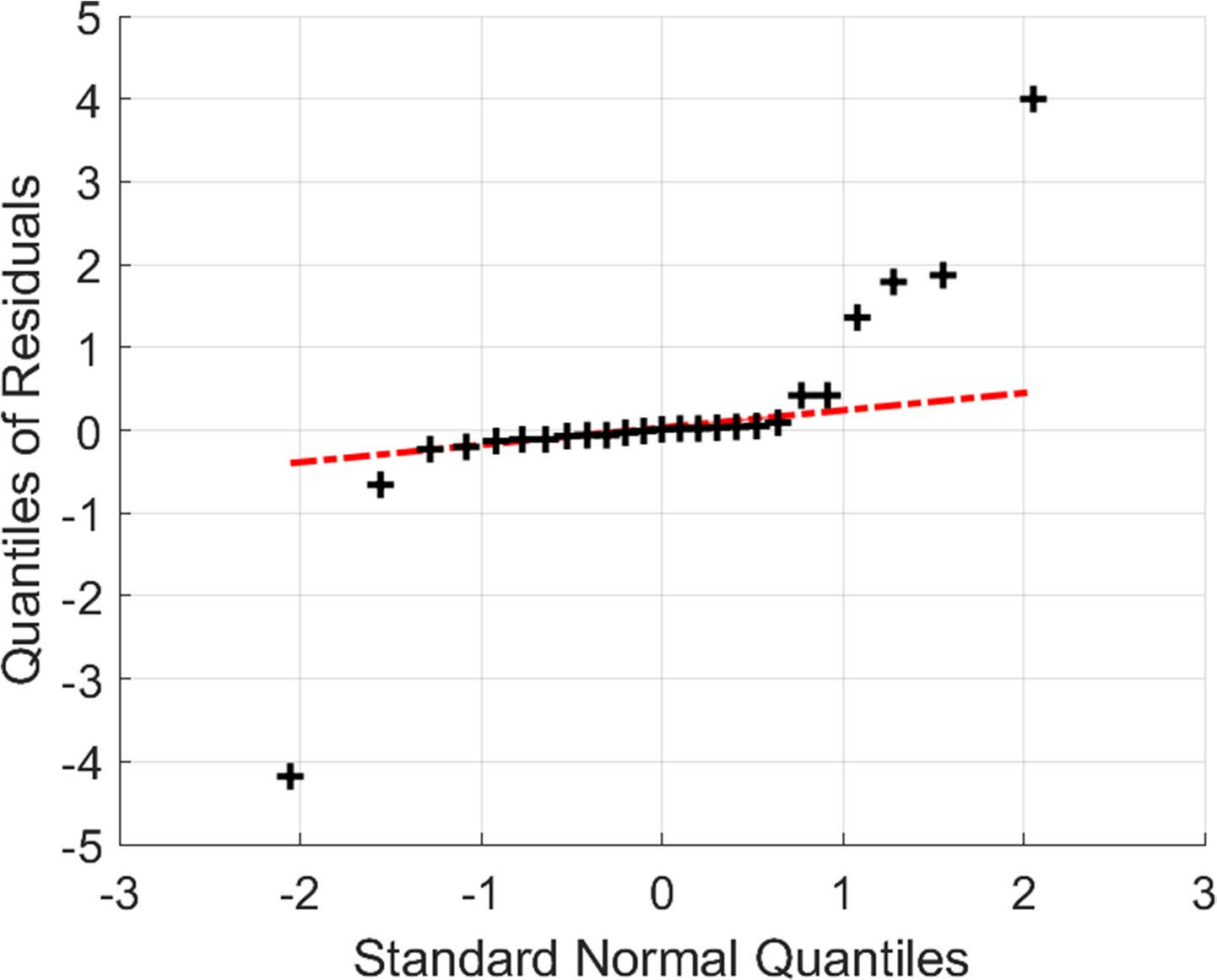
Quantile-Quantile plot of residuals from the lipase activity model (Table 4) in relation to the standard normal distribution

The experimental values, ANN-PSO predicted responses, residuals, and MSE are presented in Table 5, with experiments 13, 14, 15, and 16 expressed as the average of the central point values. In conservative approaches, the dataset used could be considered small for training a neural network [38]. However, the residual analysis shows low model bias, with residuals exhibiting a near-normal distribution with a mean of 0.17, as indicated by the quantile-quantile (Q-Q) plot in Fig. 5. Only experiments 19 to 23 deviate from the red straight line, but nearly all belong to the training set.

The MSE presented in Table 5 further supports the model’s accuracy analysis. The square root of the MSE, corresponding to an average error of 1.19, represents approximately 0.4% of the total data range (0 to 297.50). This result demonstrates that deviations between predicted and experimental values are low compared to the variable range analyzed, highlighting the model’s suitability for the proposed task.

**Table 5:** Samples divided into training, validation, and test groups with respective experimental and predicted responses for lipase activity optimization in crude extract with lipase activity (CELA)

Samples	Time (days)	Inductor (%)	Moisture (%)		U g^− 1^ Experimental	U g^− 1^ Predicted	Residuals
Train							
4	5	25		64	135.00	134.22	0.78
5	3	20		56	37.50	39.18	-1.68
6	5	20		56	105.00	105.06	-0.06
8	5	20		72	55.00	55.53	-0.53
9	4	15		56	190.00	191.91	-1.91
10	4	25		56	130.00	130.23	-0.23
11	4	15		72	57.50	58.41	-0.91
12	4	25		72	90.00	90.96	-0.96
17	2	20		64	82.50	82.40	0.10
18	6	20		64	157.50	157.21	0.29
19	4	10		64	127.50	128.61	-1.11
20	4	30		64	147.50	147.07	0.43
22	4	20		80	97.50	97.99	-0.49
23	1	20		64	35.00	34.82	0.18
26	4	35		64	172.50	172.35	0.15
27	4	20		40	0.00	0.57	-0.57
28	4	20		88	67.50	67.18	0.32
Validation							
7	3	20		72	140.00	145.52	-5.52
21	4	20		48	115.00	81.69	33.31
24	7	20		64	207.50	242.32	-34.82
25	4	5		64	297.50	286.18	11.32
Test							
1	3	15		64	85.00	81.51	3.49
2	5	15		64	120.00	116.51	3.49
3	3	25		64	62.50	47.56	14.94
13/14/15/16	4	20		64	149.42	133.80	15.62
						**MSE** ^**1**^	1.43

**Fig. 6:**
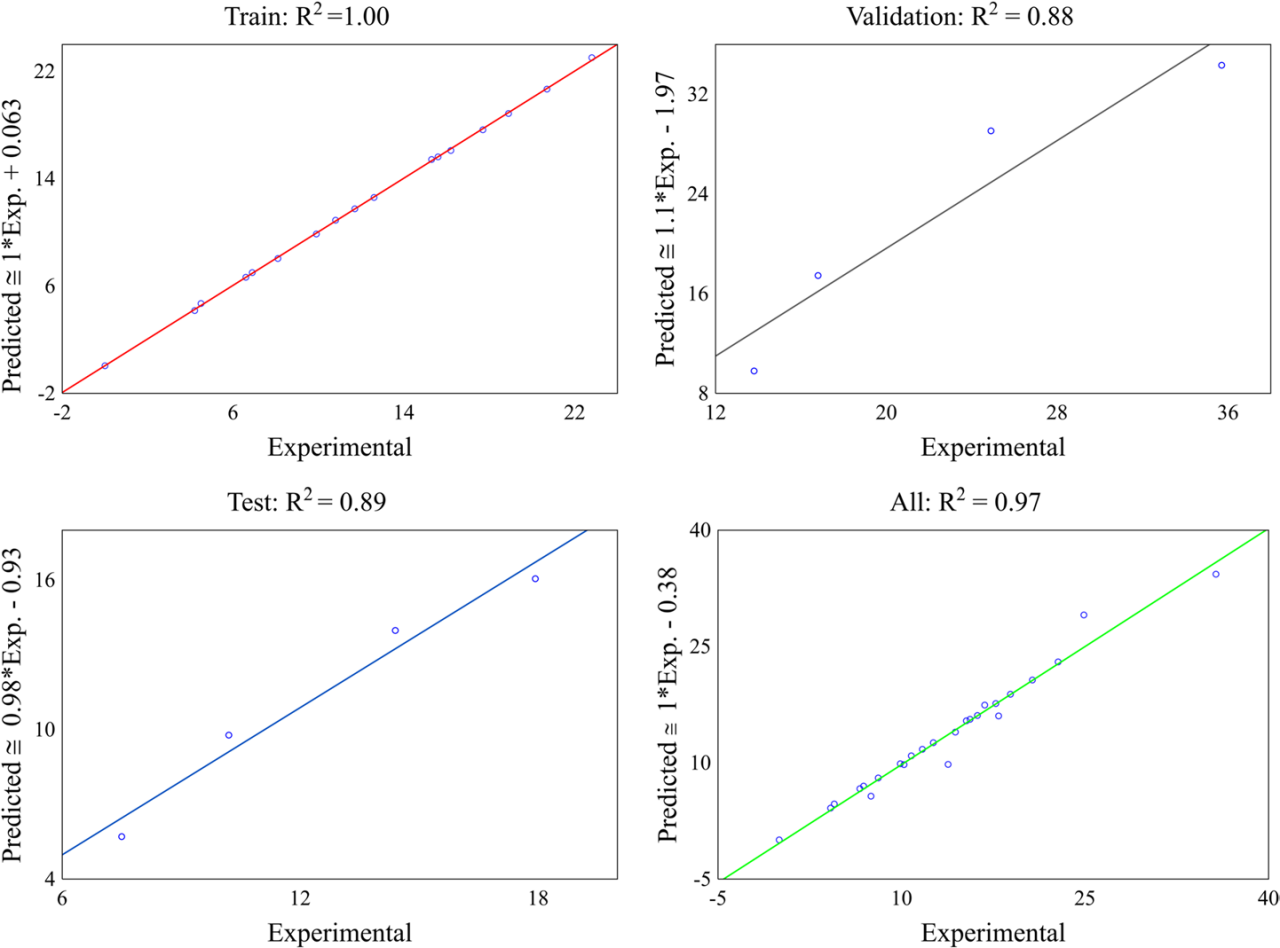
Observed vs. predicted plots for the training, validation, test, and total sets of the ANN model for lipase activity in crude extract with lipase activity (CELA), along with their respective R² values

The analysis of the observed vs. predicted plots (Fig. 6) highlights the model’s performance in each data group. The training set was used to adjust the weights and biases of the neurons, enabling the model to learn the data patterns and minimize errors.

The ANN model achieved an R² value of 0.9998 on the training data, which may indicate overfitting. However, the R² values of 0.91 and 0.97 for the validation and test sets, respectively, suggest good generalization capability, as these datasets were not directly involved in the optimization of the network parameters (Fig. 6). The reduced number of experiments, combined with the high complexity of the data (typical of experiments involving microorganisms, which exhibit significant variability in their responses) may hinder the model’s ability to capture system fluctuations [39].

When training an artificial neural network (ANN) with a large dataset, it is likely that for each example in the test or validation sets, there are similar data in the training set, facilitating the achievement of high R^2^ values across all sets. On the other hand, when the dataset is small, the problem’s representativeness is limited, which directly impacts the R^2^ values for test and validation, making them highly dependent on the specific characteristics of the modeled system [39]. This condition justifies the slightly lower-than-0.99 R^2^ values in the validation and test sets. Nonetheless, the overall performance of the model remains satisfactory, as the combined data yielded an R^2^ of 0.97, indicating strong predictive capacity and modeling consistency.

Studies reported in the literature have shown R^2^ values of 0.9994 for training, 0.8558 for test data, and 0.9648 for full data using ANN-PSO to optimize the composition of fermentation media for lipase production by *Aspergillus niger* [40]. In a study using ANN with genetic algorithms (ANN-GA) for cellulase optimization, R^2^ values of 0.99991 for training, 0.99994 for validation, and 0.99998 for testing were obtained [18]. ANN-GA applied to pectinase extraction showed R^2^ values of 0.99996 for training, 0.99787 for testing, and 0.99958 for all data [41]. ANN-GA used for lipase optimization by *Penicillium roqueforti* ATCC 10,110 reported R^2^ values of 0.99999 for training, validation, and testing [9].

Unlike these studies, which employed ANN-GA and did not consider the use of inducers in the fermentation media [9, 13, 18], the present work proposes an innovative approach by employing ANN-PSO to optimize lipase production, evaluating the effectiveness of palm oil waste as a lipid-inducing agent. Oil Palm was previously used as an inductor in a study with *P. roqueforti* ATCC 10,110 [7], but the results were limited by the use of a OFAT approach, which does not account for interaction effects between variables.

### ANN-PSO Optimization and Validation

After the modeling with the neural network, PSO was employed to determine the optimal operating conditions. Based on the established limits and the results predicted by the model, PSO generated two optimal points for the evaluated parameters: the first obtained from more restrictive search criteria and the second from a broader approach. This step was crucial for linking the model predictions with the identification of ideal experimental conditions. The obtained values were experimentally validated, enabling the assessment of the correspondence between the predictions and the actual system performance. Table 6 presents the optimal conditions generated by the ANN-PSO and ANN-GA optimization, including the predicted responses from the model and the average experimental values obtained from the triplicate experiments, corrected based on the repetition of the central point. By comparing the optimal conditions identified by PSO and GA (Table 6), it is possible to observe a clear convergence between the values, confirming the reliability and robustness of the optimization approach. This similarity indicates that the solution is not dependent on a single algorithm and suggests that the identified optimum represents a true global solution rather than a local maximum.

**Table 6:** Data obtained from ANN-PSO and ANN-GA optimization and respective experimental responses from validation for lipase activity optimization in crude extract with lipase activity (CELA)

Search region [MIN^1^ : MAX^2^]	Optimal conditions of the variables of PSO					
	Time (days)	Inductor (%)	Moisture (%)	Output ANN-PSO (U g^− 1^)		Response exp. (U g^− 1^)
Time [1 : 7] ind.^3^ [5 : 35] moisture^4^ [40 : 88]	4,24^*^	5^*^	42,4^*^	621.80		322,50 ± 4,24
Time [2 : 6] ind.^3^ [10 : 30] moisture^4^ [48 : 80]	4,37^*^	10^*^	48^*^	424.43		430.00 ± 3,31

	**Optimal conditions of the variables of AG**					
**Search region** **[MIN**^**1**^ : **MAX**^**2**^**]**	**Time (days)**	**Inductor (%)**	**Moisture (%)**	**Output ANN-AG (U g**^**− 1**^**)**		**Response exp. (U g**^**− 1**^**)**
Time [1 : 7] ind.^3^ [5 : 35] moisture^4^ [40 : 88]	4,24^*^	5^*^	42,4^*^	621.70		322,50 ± 4,24
Time [2 : 6] ind.^3^ [10 : 30] moisture^4^ [48 : 80]	4,37^*^	10^*^	48^*^	424.34		430.00 ± 3,31

^1^Minimum represents the lowest value of the search area in the ANN-PSO

^2^Maximum represents the highest value of the search area in the ANN-PSO

^3^Inductor

^4^Moisture

^*^Values of the optimal conditions predicted by the network for each variable

The analysis of the data presented in Table 6, comparing the output values of the network and the experimental response, reveals that the experiment validated the maximum value in the RSR, where the experimental value (430.00 ± 3.31 U g⁻¹) corresponds to 101.31% of the expected value at the network output (424.43 U g⁻¹), which reinforces the reliability of the model in this area. Furthermore, when comparing the experimental response with the theoretical value predicted by the quadratic model (160.83 U g^− 1^) in Section  Optimization Response Surface Methodology (RSM), the ANN-PSO optimization outperformed it by 200%. This value is very close to that obtained in the study reporting the optimization of *Penicillium roqueforti* ATCC 10,110 CELA production with ANN-GA at 101.5% of the predicted value [9]. However, the maximum value indicated in the BSR was not experimentally validated. This behavior is expected because the RSR has a higher density of experimental data, making the patterns learned by the model more robust and reliable. The larger amount of information in the experimental domain contributes to more accurate modeling, while areas with lower data density, like the BSR, are more prone to fluctuations.

**Fig. 7:**
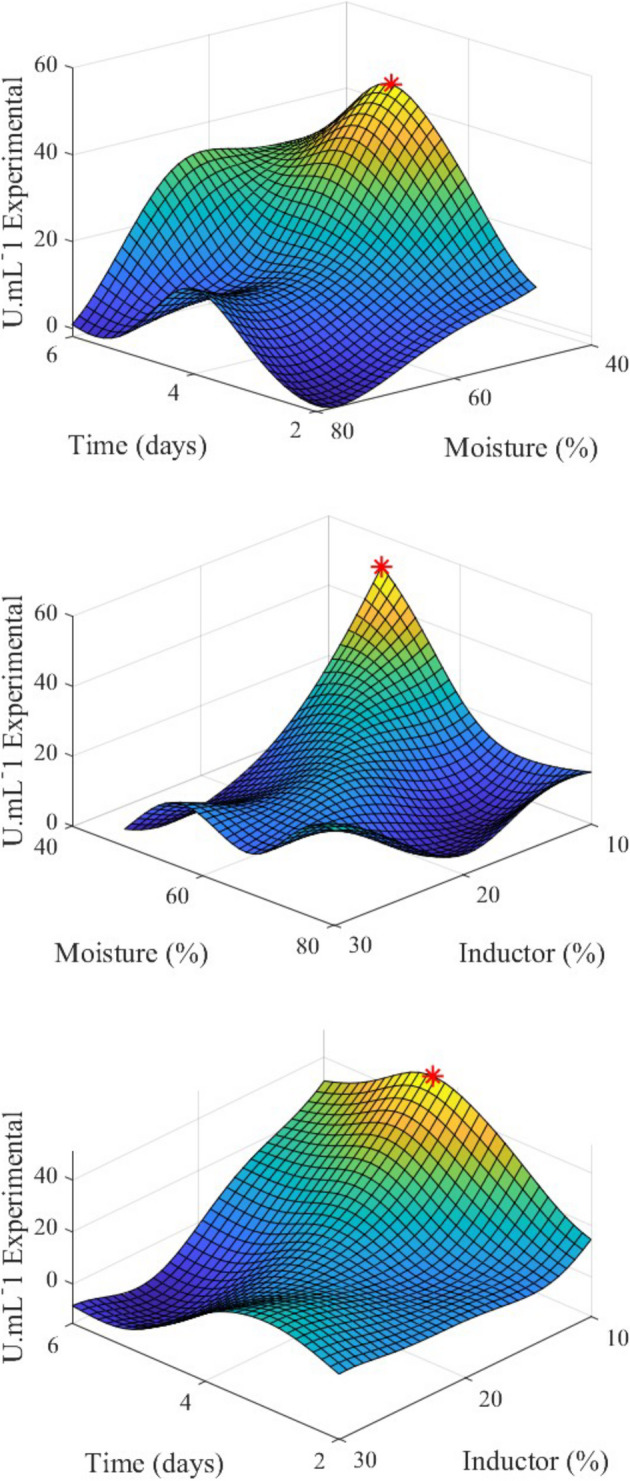
Response surface plot illustrating the effect of the evaluated variables on the Restricted Search Region (RSR) for lipase activity in crude extract with lipase activity (CELA)

**Fig. 8:**
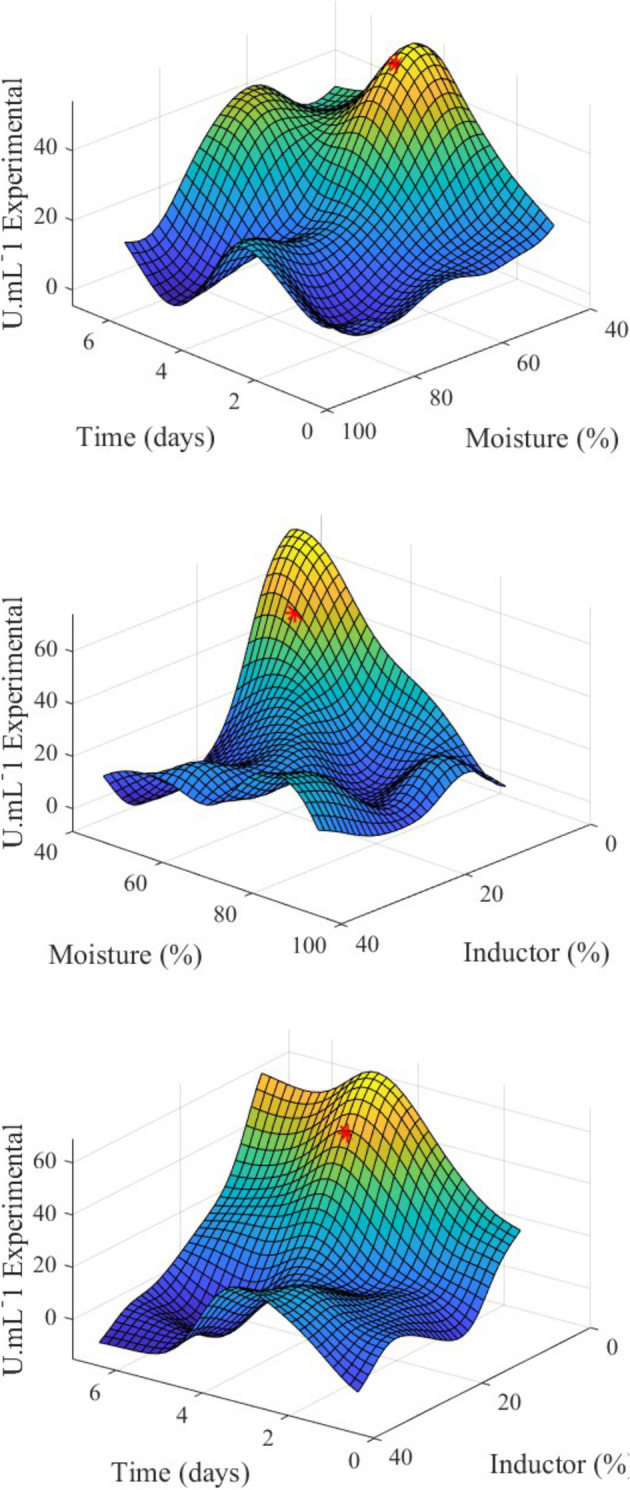
Response surface plot illustrating the effect of the evaluated variables on the Broader Search Region (BSR) for lipase activity in crude extract with lipase activity (CELA)

Furthermore, it is characteristic of MLP models trained with backpropagation to exhibit higher sensitivity at the edges of the training domain [42]. In this region, the model often performs extrapolations, as it has fewer data to support efficient weight adjustments. As a result, the prediction of the maximum in less densely sampled areas tends to be less accurate [42]. Figs. 7 and 8 show the response surfaces corresponding to the RSR and BSR, respectively, generated by ANN-PSO. It can be observed that the surfaces in Fig. 8 exhibit more pronounced fluctuations at the edges of the experimental domain, supporting the discussion on the model’s limitations in regions with less data support.

The statistical analysis of the data provided a detailed view of the model’s ability to predict and generalize the optimized process conditions. However, for a comprehensive understanding, it is crucial to discuss the microbiological aspects related to the fungus *Aspergillus niger* ATCC 1004 and the fermentation medium used. Although ANN-PSO demonstrated a high learning and generalization capability, the metabolic functioning of the fungus must be considered to explain the optimal point that was not experimentally validated. This is due to the complexities inherent in microbial metabolism, which may be influenced by biological variables not fully captured by the model.

Analyzing the optimal points generated by ANN-PSO, a marked difference is observed between the RSR point, which predicts 424.43 U g^− 1^ of lipase activity, and the BSR point, which predicts 621.80 U g^− 1^. This discrepancy is mainly attributed to differences in the variables, inductor, and moisture. In the BSR point, both variables have significantly lower values, while the fermentation time is similar between the two.

The analysis of the inductor, represented by the addition of POILS as a lipid source, highlights the importance of maintaining an appropriate balance. Very low quantities, such as the 5% planned for the BSR point, can be quickly depleted by the fungus, prompting it to seek other nutrient sources in the medium, diverting its enzymatic production towards other enzymes at the expense of lipase production [43]. On the other hand, excessive amounts of POILS can have inhibitory effects on microbial growth, possibly due to toxicity or osmotic imbalances in the fermentation medium [7]. For example, in a univariate study on the SSF of *Penicillium roqueforti* ATCC 10,110 using cocoa pod husk, the maximum lipolytic activity was achieved with 30% palm oil, stabilizing at concentrations above this level [7]. The experimentally validated optimal point in this study, with 10% inductor, demonstrates a balance that favors both growth and CELA production, confirming the need to avoid extremes in this variable.

The moisture content of the fermentation medium also plays a crucial role in fungal growth and enzyme production. Although filamentous fungi, such as *Aspergillus niger* ATCC 1004, are tolerant to low water activities, a moisture content of 42%, as indicated at the BSR point, may be insufficient to sustain efficient fungal growth. Other studies have reported that inadequate moisture limits the availability of soluble nutrients [44], which can also hinder the transport of essential substrates for lipase production. On the other hand, excessive moisture levels can reduce medium aeration, creating unfavorable conditions for the fungus’s aerobic metabolism, as reported in other studies [45, 46]. These factors reinforce the validity of the experimentally optimized values, which balance the conditions of the fermentation medium to maximize CELA production. Literature has reported that high moisture levels promote fungal sporulation but inhibit enzymatic production, while very low moisture levels inhibit fungal growth [46, 47].

The results emphasize that both the inductor and the moisture content of the fermentation medium must be carefully adjusted to balance the ideal conditions for fungal growth and CELA production. Values outside the appropriate range, whether low or high, can compromise the process’s performance, either through nutrient limitation, premature inductor depletion, or the creation of inhibitory conditions for microbial growth. This relationship highlights the importance of optimizations that consider the metabolic characteristics of *Aspergillus niger* ATCC 1004 and the interactions between the components of the fermentation medium, enabling the maximization of the system’s efficiency.

The addition of lipid-inducing sources, such as POILS, enhances CELA secretion, increasing the lipolytic activity in the fermentation medium, it has been shown that when exposed to media containing lipids, the presence of lipids and fatty acids in the fermentation medium has been shown to induce fungal lipase gene expression, including in *Aspergillus niger*, thereby enhancing lipolytic activity [40, 48, 49]. This aspect has been explored through the use of differents lipase inducers in *Aspergillus niger*. The use of 1% and 2% olive oil resulted in lipolytic activities of 182 and 172 U mL^− 1^, respectively [49]. Inducers such as Triton X-100, Tween 80, and Tween 20 led to the production of 520.95 U g^− 1^ of lipase [40]. Cacay butter as an inductor resulted in the production of 308.14 U g^− 1^ of lipase by *Aspergillus terreus* NRRL-255 [50]. The use of olive oil, castor oil, and jatropha oil resulted in the production of 48.87 U mL^− 1^ of lipase [51]. In the present study, the inducer effect of POILS was confirmed by repeating the fermentation under the optimal conditions established for the RSR, but in the absence of POILS. The results are presented in Table 7, and the statistical analysis (*p*-value < 0.05) confirms that the differences observed were significant, thus validating the positive impact of POILS on lipase activity.

**Table 7:** Effect of POILS as a lipid inducer on lipase activity (U g^− 1^ of dry substrate) under optimized fermentation conditions

	Replicates			Mean	*p*-value
CBS without POILS (U g^− 1^)	200.50	170.65	180.33	183.83 ± 15.23	0.0008
CBS with POILS (U g^− 1^)	430.21	426.70	433.32	430.08 ± 3.31	

Some of these studies used pure commercial inducers, which, although efficient, come with a high cost. For example, Triton X-100 is priced at approximately 8,650.00 U$/ton [52], while Tween 80 costs around 18,760.00 U$/ton [53], and Tween 20 are priced at about 3,860.00 U$/ton [54]. While not a full Life Cycle Assessment (LCA) or Techno-Economic Analysis (TEA), this simplified cost comparison offers an initial perspective on the potential impact of inducer prices on production costs. In contrast, the approach proposed in this study, which uses POILS as an inductor, is not only technically effective but also presents a significant economic advantage: the raw material is low-cost (in fact, a waste-derived byproduct obtained at no cost from industry), widely available, and can considerably reduce input expenses.

The same discussion applies to the solid medium used in this study: CBS, a byproduct that is often undervalued [55]. Its use as a raw material for CELA production will result in CBS impregnated with *Aspergillus niger* biomass. Since this fungus is classified as GRAS, it opens up the possibility of valorizing the resulting solid phase as a food ingredient or nutraceutical, which could be explored in future studies. Furthermore, after the inactivation of this biomass, its use as a biofertilizer can be investigated, adding significant value to the material and expanding its potential for sustainable use while supporting a circular economy approach.

Additionally, the use of crude enzymatic extracts, such as those produced in this study, derived from agro-industrial residues offers clear advantages in terms of cost reduction and process simplicity, especially for applications where enzyme purity is not critical [11, 56]. In such cases, the crude extract can be directly applied to biocatalytic processes, such as transesterification for biodiesel production [11], biodegradation of polyester [56], wastewater treatment [57] and saccharification of agro-residues [58], where the presence of non-enzymatic components may not significantly interfere. Futhermore, the patent BR 112013005916-8 B1 describe generating enzymatic formulations from agro-industrial waste utilizing SSF, like corn starch or sugarcane bagasse, to efficiently convert biomasses rich in starch and lignocellulose into fermentable sugars [59]. However, for applications requiring high specificity and high purity in the products (such as food or pharmaceutical products), downstream processing becomes essential. Techniques such as ultrafiltration, precipitation, crystallization, or immobilization can be employed to partially purify or stabilize the enzymatic preparation [60].

## Conclusion

The effectiveness of the ANN-PSO approach was demonstrated in solving problems with limited data. By utilizing just 28 experiments, the method successfully optimized an ANN without requiring modifications to the experimental domain or additional practical trials, leading to savings in reagents and analysis time. Experimental validation confirmed the accuracy of the algorithm’s predictions, achieving 101% of the expected enzymatic activity, underscoring the effectiveness of the methodology. POILS showed potential as inducer in the lipase activity in the crude fungal extract via SSF.

The ANN-PSO approach significantly contributes to the rapid development of potentially sustainable bioprocesses, paving the way for future biotechnological applications and optimizations in SSF systems. Future research should explore potential pathways for expanding the process beyond the laboratory scale and propose solutions for the management of the solid phase resulting from CELA production, building on the approach applied in this study.

## Data Availability

The data that support the findings of this study are available from the corresponding author upon reasonable request.

## Declarations

### Submission Declaration

The results/data/figures in this manuscript have not been published elsewhere, nor are they under consideration by another publisher.
